# Skin Metastasis of Renal Cell Carcinoma: A Case Report

**DOI:** 10.7759/cureus.3614

**Published:** 2018-11-19

**Authors:** Murat F Ferhatoglu, Kazim Senol, Ali I Filiz

**Affiliations:** 1 General Surgery, Okan University, Istanbul, TUR; 2 General Surgery, Koc University Hospital, Istanbul, TUR

**Keywords:** metastasis, renal cell carcinoma, skin

## Abstract

Metastasis of renal cell carcinoma is seen in approximately 25% of all cases. Rarely, they can appear in unusual sites. Herein, we present a 40-year-old female patient with an itching scalp mass. The mass appeared one year after the nephrectomy performed for a right renal cell carcinoma. Computed tomography scans have not identified any metastasis during the postoperative evolution of the disease. We excised the mass with a large surgical margin under local anesthesia. Pathological examination of the lesion diagnosed metastasis of clear cell carcinoma. Our case is not just the rare metastatic site but also the fact that the tumor appeared despite its low grade (T2N0MO). Unfortunately, the prognosis of metastatic renal cell carcinoma* *(RCC) with skin metastasis is in most cases unfavorable. We believe that our case could add more information to the following measures, complete the frame of rare oncologic cases and consolidate the data published on the topic so far. Although skin metastases are a poor sign of progression, disease-free follow-up is possible after appropriate surgical excision.

## Introduction

Renal cell carcinoma (RCC) accounts for 2–3% of all solid cancers, usually occurs between fifth and seventh decades of life and is twice as common in males [1,2]. Approximately 30% of cases metastasize at the time of admission [3]. Metastases frequently occur in the lungs, liver, and bones [4]. However, skin metastasis is a rare entity. In this article, we present a woman diagnosed with scalp metastasis stemming from RCC one year after the operation.

## Case presentation

**Figure 1:**
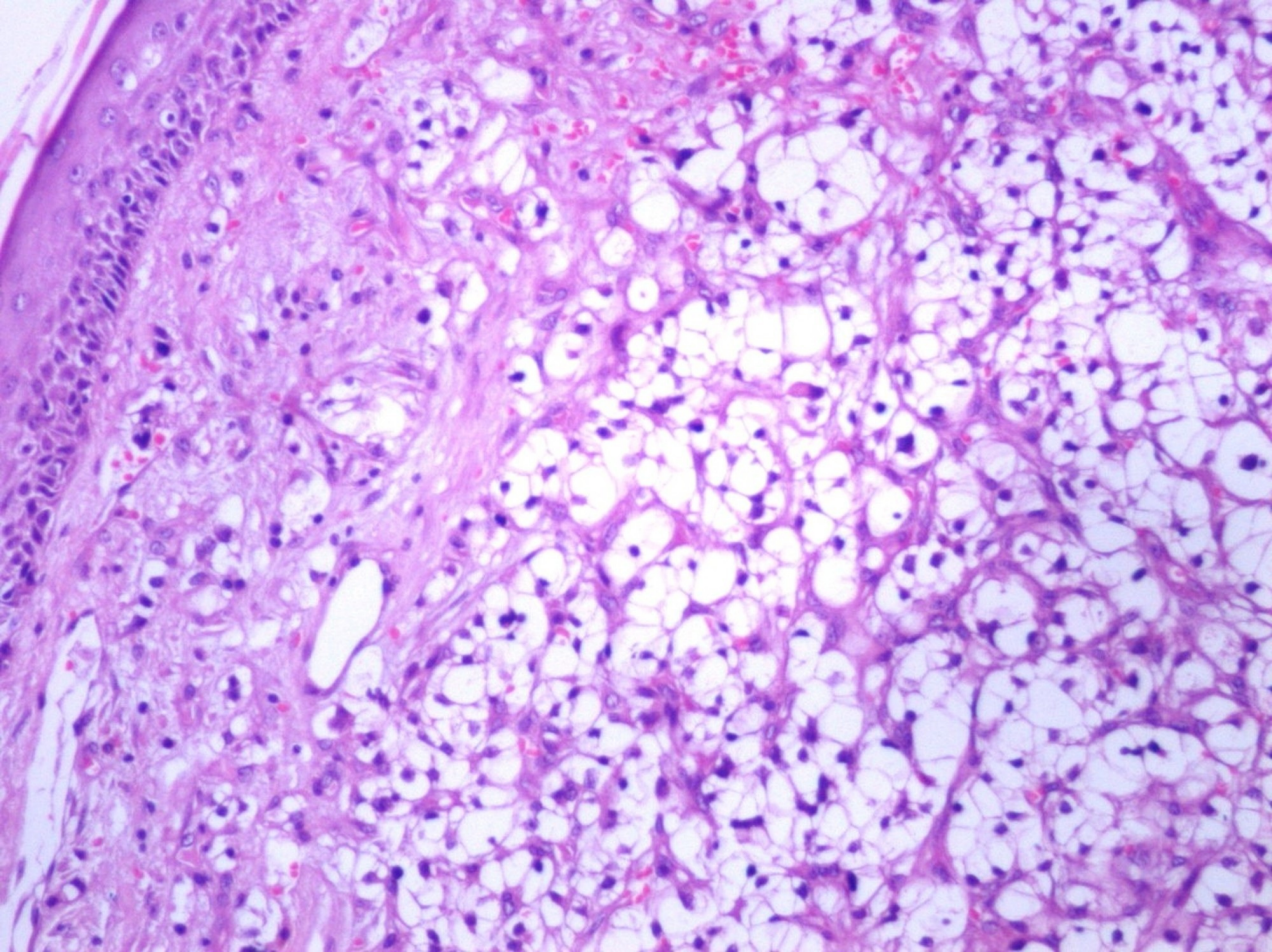
Renal cell carcinoma, hemotoxylic section x200 HPF.

**Figure 2:**
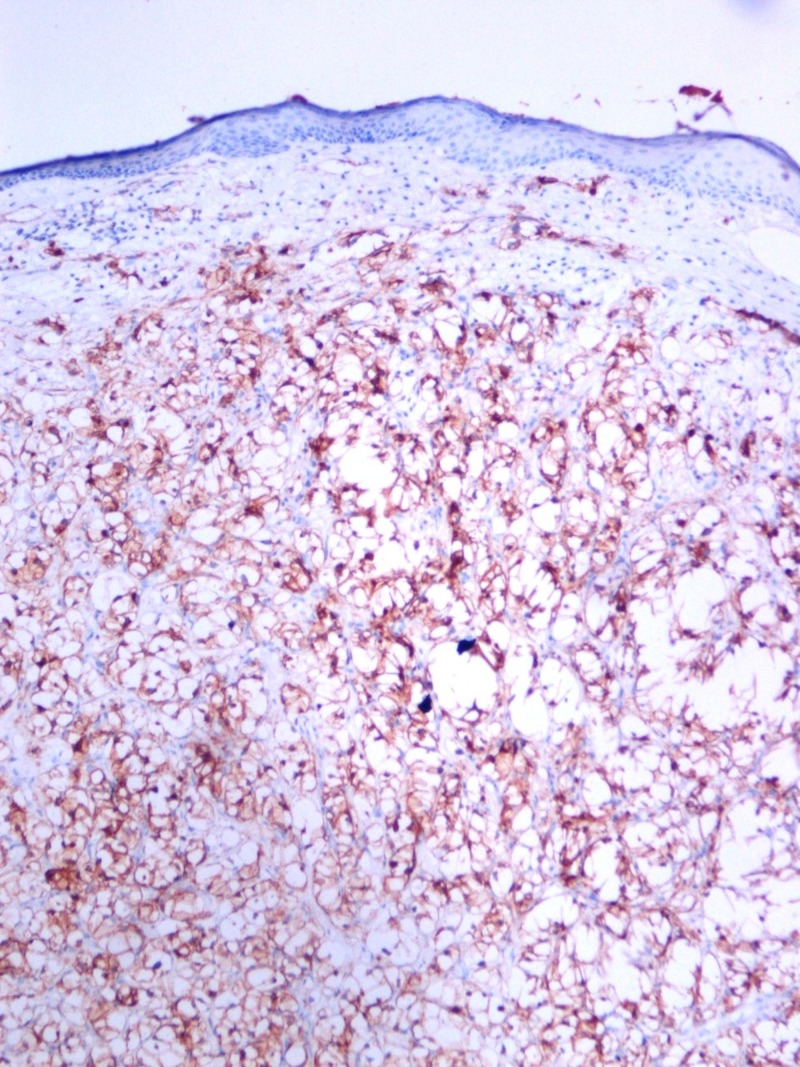
Renal cell carcinoma immunohistochemistry, CD10, x100 HPF.

**Figure 3:**
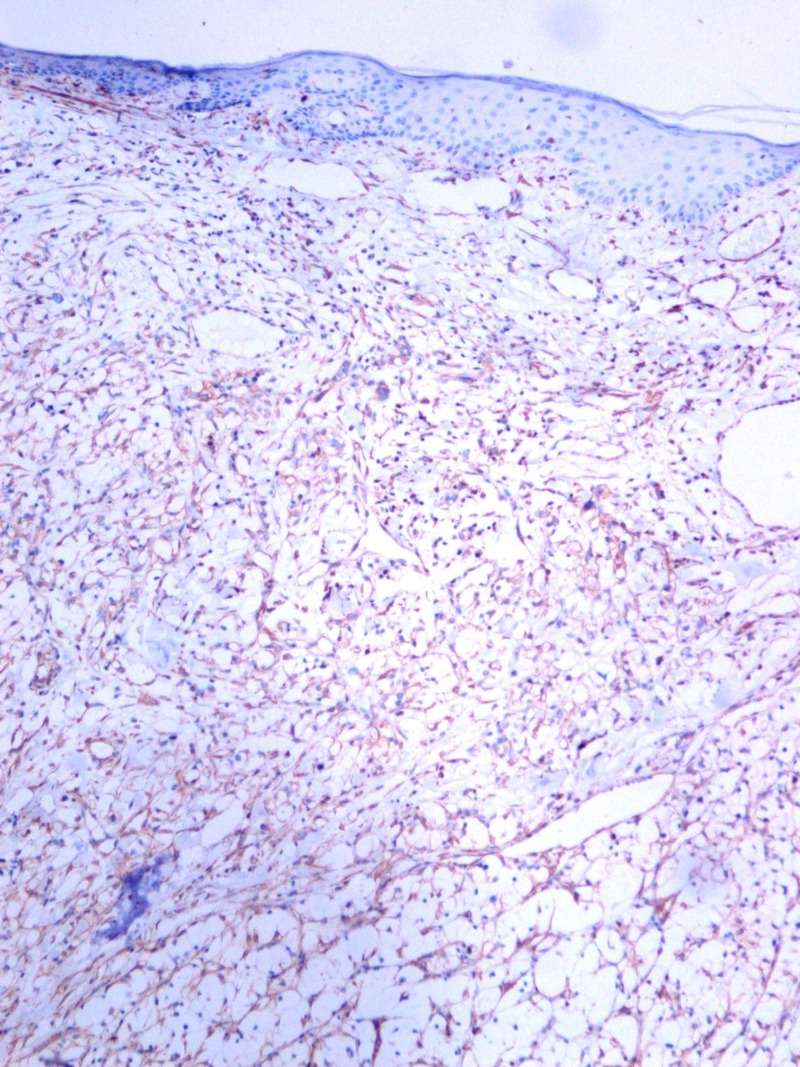
Renal cell carcinoma immunohistochemistry Vimentin, x100 HPF.

**Figure 4:**
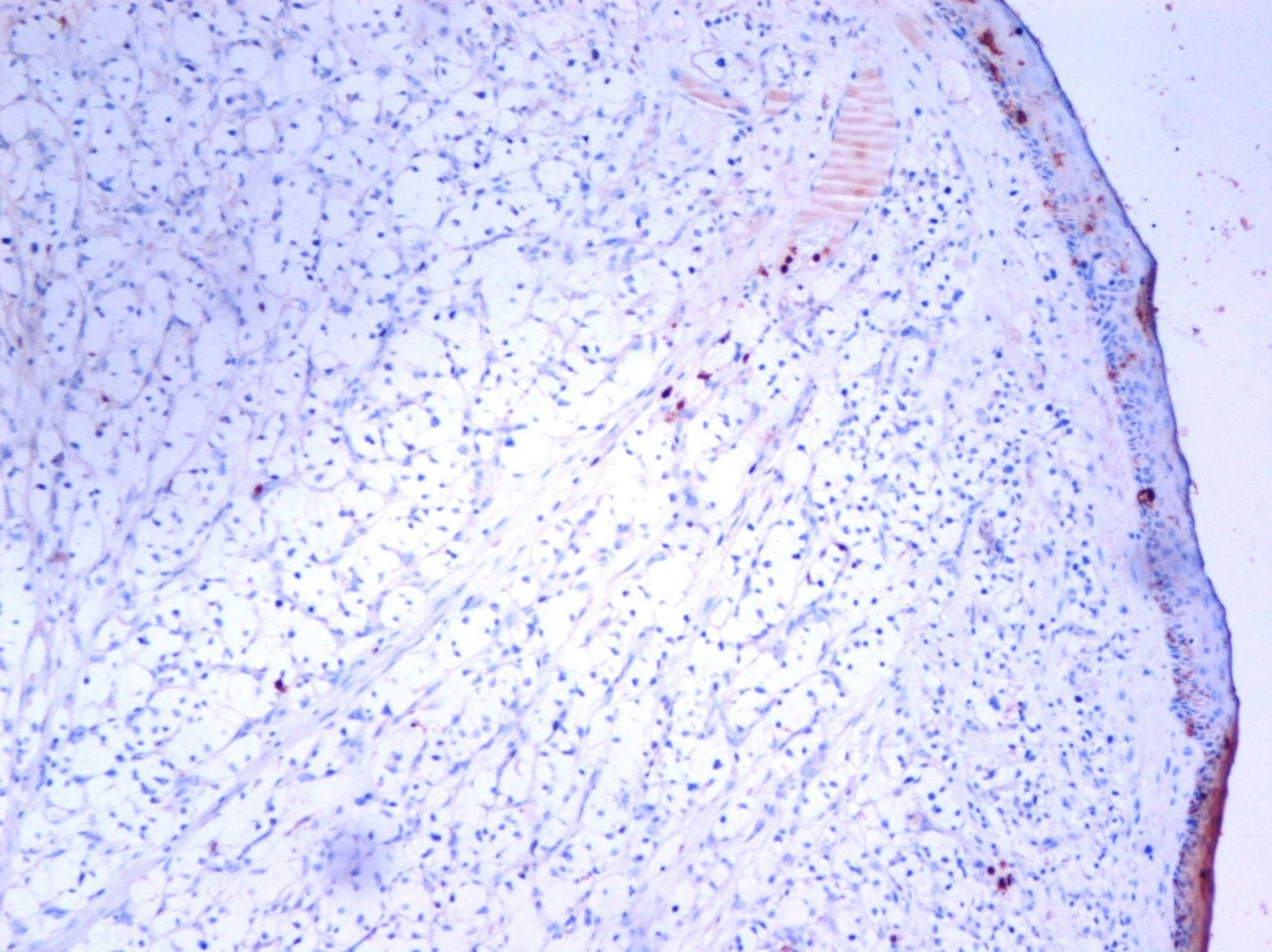
Renal cell carcinoma immunohistochemistry, S100, x100 HPF.

**Figure 5:**
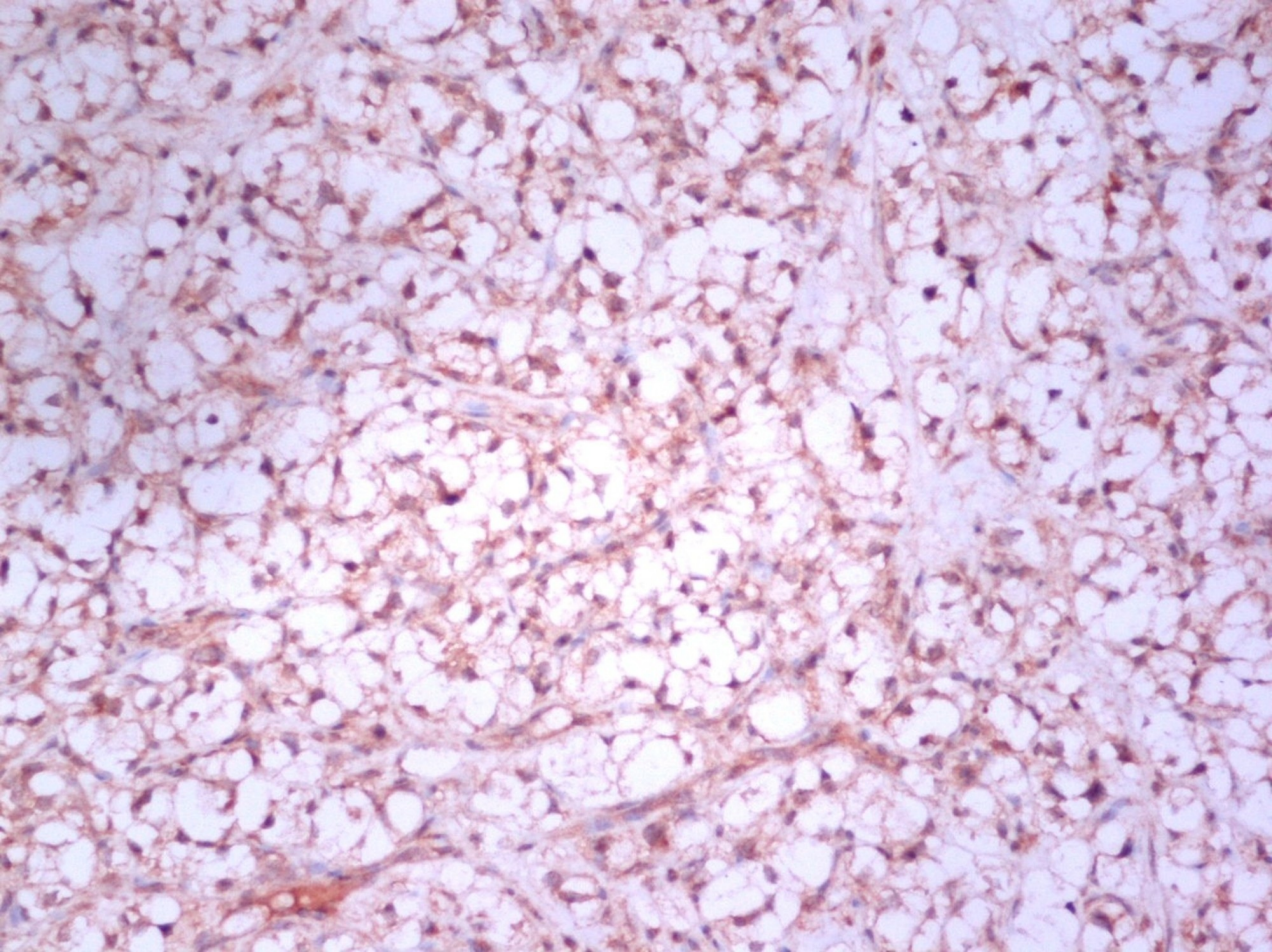
Renal cell carcinoma immunohistochemistry RCC dye, x100 HPF.

**Figure 6:**
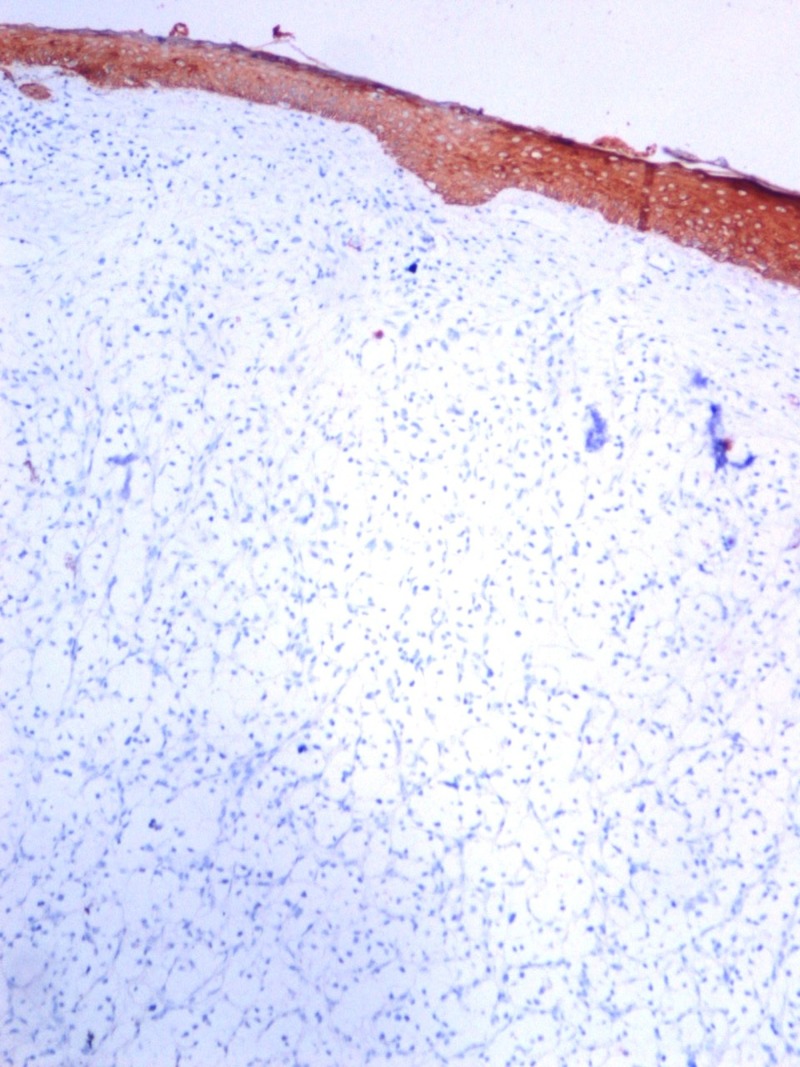
Renal cell carcinoma immunohistochemistry Pan-CK, x100 HPF.

A 40-year-old woman presented with an itching mass that was found three weeks ago on the head. In her history, she was operated because of renal cell carcinoma (T2, N0, M0) 14 months before. There was no other known disease, and she had no problem in the routine follow-up. On physical examination, we found a smooth, red-colored, well-defined mass, 0.5 cm in diameter on the occipital region of the scalp. Local excision was decided because of a newly emerging lesion and discomfort to the patient. We excised the mass with a large surgical margin under local anesthesia. The lesion was diagnosed as clear cell carcinoma in the pathological examination (Figure 1) and evaluated as renal cancer metastasis. The tumor existed with 4 mm surgical margin. Immunohistochemically, the lesion was positive for CD10 (Figure 2), vimentin (Figure 3), and negative for S100 (Figure 4) renal cell carcinoma dye (Figure 5), pan-cytokeratin (Figure 6); CD34, CEA, HBM45. No metastasis was detected elsewhere on the patient's scans. In the first year after the metastasectomy, the patient is followed without any problems.

## Discussion

Renal cell carcinoma is responsible for about 3% of adult tumors. The classic triad of renal cell carcinoma is palpable mass, hematuria and back pain. However, only 10% of the patients have these three findings together [5]. On presentation, it was learned that about one year ago, renal cell carcinoma was diagnosed after the complaints of back pain and hematuria.

Renal cell carcinoma often metastasizes to the lungs, liver, bones, lymph nodes, counter kidney or adrenal glands [6]. The metastatic skin lesion is a rare entity wand seen in only 2.8–6.8% of the patients [2]. A total of 80–90% of patients with skin metastases are patients with a prior diagnosis of renal cell carcinoma. However, 10–20% of patients are diagnosed with skin lesions before the primary lesion is identified [2]. Skin metastasis of renal cell carcinoma most commonly observed on face and scalp [6]. Lesions usually occur between six months and five years after the first diagnosis. Another distant metastases or recurrence of the tumor are found in the majority of patients [7]. In our case, skin metastasis was detected 14 months after the first diagnosis, and no other metastatic focus or recurrence was detected. RCC skin metastasis is often a poor prognostic indicator, and the expected lifespan is less than six months [4]. The presented case has survival without disease at the end of the first year of skin metastasectomy.

Skin metastases of renal cell carcinoma present as nodular, rapidly growing, round or oval-shaped lesions, which can be of various colors ranging from normal skin color to a red-purple color [8]. Clinical presentation may be confused with hemangioma, basal cell carcinoma or pyogenic granuloma [1]. There was a similar appearance of hemangioma in our case. In histopathological examination, atypical nucleated cells are expected to be seen in clear cell type. The nodular mass is surrounded by the atrophic epidermis, and moderate lymphocytic infiltration can be observed [8]. Lesions should be considered xanthoma, xanthelasma, hidradenoma in the differential diagnosis. The immunohistochemical examination provides a microscopic differential diagnosis. Epithelioid membrane antigen, carcinoembryonic antigen, CD-10, renal cell carcinoma marker are markers used to identify skin metastases of renal cell carcinoma [9,10].

Metastatic renal cell carcinoma therapy consists of surgical (radical nephrectomy) treatment and the combination of angiogenesis/multikinase inhibitors (sunitinib, sorafenib) [6]. The treatment approach for single, isolated skin lesions is surgical removal of the lesion only. Radiotherapy may be an alternative to surgery in cases where surgical intervention is not feasible [6]. Our patient did not receive any additional treatment except extensive surgical resection of the skin lesion.

## Conclusions

Newly occurring skin lesions of renal cell carcinoma patients should be carefully evaluated. Although skin metastases are interpreted as a bad sign of progression, disease-free follow-up is possible after appropriate surgical excision.
